# Controlling the Multifractal Generating Measures of Complex Networks

**DOI:** 10.1038/s41598-020-62380-6

**Published:** 2020-03-26

**Authors:** Ruochen Yang, Paul Bogdan

**Affiliations:** Ming Hsieh Department of Electrical and Computer Engineering, University of Southern California, Los Angeles, CA 90089 United States

**Keywords:** Computer science, Statistics

## Abstract

Mathematical modelling of real complex networks aims to characterize their architecture and decipher their underlying principles. Self-repeating patterns and multifractality exist in many real-world complex systems such as brain, genetic, geoscience, and social networks. To better comprehend the multifractal behavior in the real networks, we propose the weighted multifractal graph model to characterize the spatiotemporal complexity and heterogeneity encoded in the interaction weights. We provide analytical tools to verify the multifractal properties of the proposed model. By varying the parameters in the initial unit square, the model can reproduce a diverse range of multifractal spectrums with different degrees of symmetry, locations, support and shapes. We estimate and investigate the weighted multifractal graph model corresponding to two real-world complex systems, namely (*i*) the chromosome interactions of yeast cells in quiescence and in exponential growth, and (*ii*) the brain networks of cognitively healthy people and patients exhibiting late mild cognitive impairment leading to Alzheimer disease. The analysis of recovered models show that the proposed random graph model provides a novel way to understand the self-similar structure of complex networks and to discriminate different network structures. Additionally, by mapping real complex networks onto multifractal generating measures, it allows us to develop new network design and control strategies, such as the minimal control of multifractal measures of real systems under different functioning conditions or states.

## Introduction

Technological advances and information digitization contribute not only to richer complex multi-modal heterogeneous and noisy datasets in diverse fields such as social, geoscience, brain and biological networks^1,2^, but at the same time call for advanced mathematical techniques for mining and investigating complex multiscale and spatiotemporal relationships. Much of the complex network literature focuses on developing mathematical network models that characterize one or few pairwise interactions^3–6^. More recently, several network modelling approaches study the network complexity from a geometrical perspective^7–12^.

Since, in various settings, we deal with networks with unknown rules of network growth and dynamic evolution (also referred as non-stationary interactions), these approaches prove beneficial to characterize some new network properties. However, in majority of the real-world experiments, we only have access to partial observations, or we notice that the interaction rules are highly heterogeneous across space and time. Modelling a system with such limitations requires a new mathematical formalism that can not only extract the generalized geometric signatures across scales, but also provide tools for scaling analysis of motifs and patterns. Additionally, the framework is desired to enable new control strategies that go beyond node state and target geometric characteristics of the network (e.g., network curvature, multifractal spectrum).

From a geometrical perspective, many large-scale complex networks from sociology and biology exhibit self-similar and multifractal characteristics^13,14^. Multifractal geometric analysis makes it possible to capture the heterogeneous and multiscale interaction rules of large networks^15,16^. It efficiently characterizes large-scale complex systems^16^ and can be employed to measure nodes similarity^17^ and detect community structures^18^. For instance, the multifractality of geochemistry mapping explains the element concentration values distribution and spatial covariance structure in rock samples^19^. In multifractal formalism, the renormalization procedure of coarse-graining networks into boxes (i.e., box-counting method) helps at examining the existence of scaling and heterogeneity of motifs and rules in networks. The self-similar exponent is defined by the renormalization and a noticeable power-law relationship is found between the number of boxes needed to cover the nodes and the scale of boxes^18,20^.

Building on the idea of self-repeating structures, the de Wijs model^21^ partitions a unit segment into two subsegments of equal size and further repeats this operation recursively, which leads to the Mandelbrot multifractal^22,23^. The model construction from multiplicative cascade results in the discrete log-binomial distribution^19,24–28^. In contrast, the stochastic Kronecker graph represents a random graph obtained from the Kronecker product of probability matrices, thus displaying a self-similarity. It is able to reproduce many properties in real networks including heavy tail degree distribution, low diameter and densification power law^29–31^. Along the same lines, the multifractal network generator generates random graphs with a wide variety of statistical properties^32,33^. The multiplicative attribute graph generalizes the stochastic Kronecker graph and the multifractal network generator^34,35^. By characterizing the interaction between nodes with different attributes, it models network structures as well as node property and information.

Prior works on modelling self-similar networks focus on unweighted graphs. However, as demonstrated in the literature^7^, much of the complexity is encoded in the heterogeneous weights of the interactions. Pure topological models are binary and they are inadequate to capture complicated properties of real complex networks^36^. In lots of situations, we deal with large complex networks where the interactions are heterogeneous and possibly varying. Introducing weights/strength can ameliorate existing binary random models to better depict and understand networks in real systems. Furthermore, there is a lack of analytical multifractal analysis for random graphs. Box-covering methods over graphs is equivalent to graph coloring problem, which is a known NP-complete problem. It’s even more difficult to apply this node-based analysis on random graphs. Moreover, we do not only want to estimate and analyze network multifractality, but also want to control the multifractality and reflect the performance of robustness and resilience in complex networks.

To address these research gaps, in this paper, we propose the *weighted multifractal graph* (WMG) model. The WMG model captures and generates weighted multifractal networks by mapping from recursively constructed measures of linking probability. Rather than covering graph nodes with minimum number of boxes, we analyze and analytically quantify the multifractal properties of networks via counting edges that satisfy similar generating rules. We analytically show the multifractal properties of the WMG model. The WMG model could produce and account for a wide variety of multifractal spectrums with varying degrees of symmetry, locations, support and shapes. It can be employed to model the complexity of a wide array of networks from brain, genomics, proteomics, social and geoscience disciplines. The benefits of the WMG formalism is twofold: (1) It helps to recover mathematical models which are capable to discriminate between different structures of a complex system. (2) It enables us to understand and control the multifractality of complex networks with minimal intervention. It provides comprehension on how to explore the mechanism of evolving networks and how to regulate complex networks under different states.

## Results

### Weighted multifractal graph model

In order to generate weighted networks with multifractal characteristics and to minimally control complex networks, we introduce the *weighted multifractal graph* (WMG) model to generate and capture random weighted graphs with multifractal topology. By choosing a few parameters, the model can generate a wide variety of weighted multifractal topologies with arbitrary statistical properties, such as generalized degree distribution and clustering coefficient. This multifractal model can be used to fit diverse real-world datasets in fields such as biological systems, geoscience and financial markets. Also, it enables to control multifractality of networks with mild modifications and adjustments.

Along the same lines as in the multifractal network generator^32^, we generate the WMG model recursively from simple square structures. First, we define simple generating measures on *R*-layer unit squares. The x-axis and y-axis of each layer are identically divided into *M* intervals, and each interval length is denoted as *l*_*i*_, *i* = 1…*M*. The unit square is divided into *M*^2^ rectangles, each rectangle (*i*, *j*) is assigned with a set of probabilities *p*_*ij*_(*r*), *i*, *j* = 1…*M*, *r* = 1…*R*. The WMG model is developed from this initial generating measure. The self-similarity appears when we divide each rectangle in the unit square into *M*^2^ sub-rectangles, which has the same structure as the unit one. The self-similar WMG model is formed after repeating the operation *K* times. Meanwhile, the assigned probabilities ${p}_{ij}^{(K)}(r)$ and interval lengths ${l}_{i}^{(K)}$ in the WMG model are the product of probabilities and interval lengths in every division. The expression of ${p}_{ij}^{(K)}(r)$ and ${l}_{i}^{(K)}$ can be written as

1 $${p}_{ij}^{(K)}(r)=\mathop{\prod }\limits_{q=0}^{K-1}{p}_{{i}_{q}{j}_{q}}(r),$$

2 $${l}_{i}^{(K)}=\mathop{\prod }\limits_{q=0}^{K-1}{l}_{{i}_{q}},$$

where ${i}_{q}=\lfloor \frac{i-1}{{M}^{q}}\rfloor \,{\rm{mod}}\,\,M+1$, ${j}_{q}=\lfloor \frac{j-1}{{M}^{q}}\rfloor \,{\rm{mod}}\,\,M+1$, and ‘mod’ represents the modulo operation. Of note, the values of *l*_*i*_ are identical for all layers. The *l*_*i*_ values are independent of the weight layer index *r*. Furthermore, this generating measure is defined on unit squares and satisfies ${\sum }_{{i}_{0}=1}^{M}{l}_{{i}_{0}}=1$ and ${\sum }_{i=1}^{{M}^{K}}{l}_{i}^{(K)}=1$.

When generating a random multifractal graph, we first select a discrete weight set {*w*(*r*)}_*r*=1:*R*_. The *r* and *w*(*r*) are the index of the discrete weight level and the corresponding weight value, respectively. ${p}_{ij}^{(K)}(r)$ and ${l}_{i}^{(K)}$ are the corresponding generating measures of the model at *r*-th layer. The weight set ${\left\{w(r)\right\}}_{r=1:R}$ can be any desired positive and real values according with specific distributions for specific (desired) network features. Here *R* is the resolution level of the discrete weights. Choosing larger *R* leads to higher graph resolution and more parameters ${\left\{{p}_{ij}(r)\right\}}_{r=1:R}$ will be needed to be introduced. Like De Wijs model^24,25^, Kronecker random graph^29–31^, stochastic block model^34^ and multifractal network generator^32^, a weighted random graph with *N* nodes is generated from the linking probability measures using the following strategy: (1) Generate a series of uniformly distributed random variables *U*(*n*) for each node *n* = 1…*N* independently. (2) Find the category *i*(*n*) of each node *n* such that ${\sum }_{t=1}^{i(n)-1}{l}_{t}^{(K)} < U(n)\le {\sum }_{t=1}^{i(n)}{l}_{t}^{(K)}$. In the recursive model with *K* iterations, this suggest that the node *n* falls into sub-interval *i*(*n*) on the unit edge. Since *l*_*i*_ and ${l}_{i}^{(K)}$ are identical for all layers, the categories *i*(*n*) assigned for the node *n* in every weight layer are also identical. (3) For each pair of nodes (*u*, *v*), generate an uniformly distributed random variable *L*(*u*, *v*), and find index *r* such that it satisfies ${\sum }_{t=1}^{r-1}{p}_{i(u)i(v)}^{(K)}(t) < L(u,v)\le {\sum }_{t=1}^{r}{p}_{i(u)i(v)}^{(K)}(t)$. Next, we assign the weight value *w*(*r*) to the link between nodes *u* and *v*. If there is no *r* satisfying this condition, then there will be no edge between this pair of nodes (*u*, *v*). It is noteworthy that when *M*^*K*^ ≈ *N* and ${\left\{{l}_{i}\right\}}_{i=1:M}$ is equally sized, if each node *n* corresponds with one category *i*(*n*) without repetition, this special case of WMG model would reduce to Kronecker graph^29–31^.

**Figure 1:**
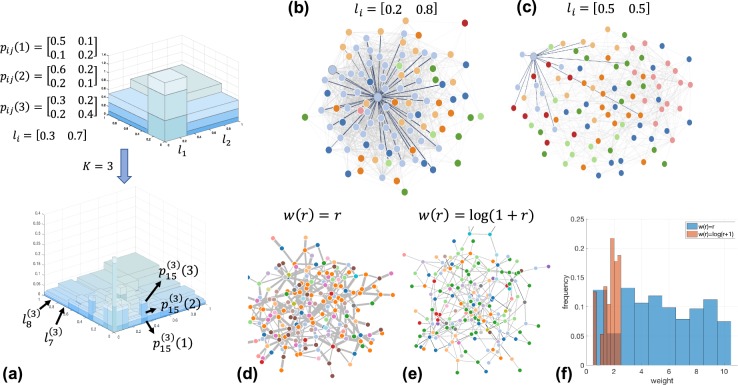
(**a**) Schematic illustration of a simple recursive WMG model generation with *M* = 2, *R* = 3. (**b**) A network generated with model interval length *l*_*i*_ = [0.2 0.8]. (**c**) A network generated with model interval length *l*_*i*_ = [0.5 0.5]. (**d**) A network generated with weight distribution *w*(*r*) = *r* for *r* = 1…10. (**e**) A network generated with weight distribution $w(r)={\rm{\log }}\,(1+r)$ for *r* = 1…10. (**f**) Normalized histogram of the weights in (**d**,**e**).

The iterative WMG model building procedure is illustrated in Fig. 1(a). The presented model example has 3 layers. In this example, $$\left\{{l}_{i}^{(K)}\right\}=\left[\begin{array}{cccccccc}0.027 & 0.063 & 0.063 & 0.147 & 0.063 & 0.147 & 0.147 & 0.343\end{array}\right]$$is built from Eq. (2) with $$\left\{{l}_{i}\right\}=\left[\begin{array}{cc}0.3 & 0.7\end{array}\right]$$. Two networks in Fig. 1(b,c) are generated under the same model parameters *M* = 2, *R* = 2, *K* = 3, $${p}_{ij}(1)=\left[\begin{array}{cc}0.9 & 0.3\\ 0.3 & 0.3\end{array}\right]$$, $${p}_{ij}(2)=\left[\begin{array}{cc}0.1 & 0.2\\ 0.2 & 0.9\end{array}\right]$$and $$w(r)=\left[\begin{array}{cc}1 & 2\end{array}\right]$$. The color of nodes represents generating category and the stroke width of edges displays the weight. We highlight one node in each graph, its neighbours and the edges connecting them. As shown in Fig. 1(b), 45 of 100 nodes are colored with light blue and they are in the same category *i* = 8 because ${l}_{8}^{(3)}=0.512$. The neighbours of the highlighted node in the middle are more likely to have the same category (34 of 45 nodes, category *i* = 8 in light blue). As a result of the relatively large value of ${p}_{88}^{(3)}(2)=0.729$, the edges connecting them have weight *w*(2) = 2. By contrast, in Fig. 1(c), the categories of nodes are more evenly distributed because the interval lengths are ${l}_{i}^{(3)}=0.125$ for *i* = 1. . . 8. The highlighted node in Fig. 1(c) has category *i* = 1. In this example, ${p}_{1i}^{(3)}(1) > {p}_{1i}^{(3)}(2)$ for *i* = 1. . . 8. Therefore, in this generated graph, all the links containing the highlighted node have weight *w*(1) = 1. Fig. 1(d,e) show two networks generated by different weight distributions *w*(*r*) = *r* and $w(r)={\rm{\log }}\,(1+r)$. While all other parameters are kept the same, from Fig. 1(d,e), we can see that a change in w(r) from *r* to ${\rm{\log }}\,(r+1)$ leads to networks with different weight features. In Fig. 1(f) we show the normalized histogram of the weights in two networks (d,e). Next, we present the multifractal analysis of this WMG model.

### Multifractal analysis

Traditional approaches for quantifying the multifractal properties of networks rely on box-counting and box-covering method^18,20,37^. The fractal dimension can also be obtained with the help of closeness centrality^38^. However, finding the minimum number of boxes of a given radius required to cover the graph is as hard as the graph coloring problem^20^. Introducing randomness in the graph model brings uncertainty to the box-counting problem. Although there exist few analytical results that determine inequality constraints on the chromatic number for the graph coloring problem in Erdős-Rényi model^39–41^, these inequalities cannot be analytically extended to networks with higher-order correlations and so determine their box covering.

To elucidate the multifractal properties of the above-mentioned weighted network construction strategy, we describe next an analytical framework capable of estimating the partition function, the mass exponent, the generalized fractal dimension, the Lipschitz-Hölder exponent and the multifractal spectrum. Since the link generating process contains both node attributes and edge formation characteristics, the proposed mathematical formalism estimates the number of edges which are generated under the same rule. While this formalism bears some similitudes with the multiplicative cascade model^26–28^, it counts the number of edges in the generated graph rather than counting sub-blocks with same structure. Our edge-based strategy enables us to analytically compute the partition function and derive the multifractal related metrics (e.g., generalized fractal dimension, multifractal spectrum). For simplicity, let ${\left\{{p}_{ij}(r)\right\}}_{i,j=1:M}$ denote the edge generating probability in the initial unit square model and ${\left\{{l}_{i}\right\}}_{i=1:M}$ denote the length of the intervals, respectively. With these conventions, the derivation of the partition function reads:

3 $${Z}_{\epsilon }(q)=\mathop{\sum }\limits_{r=1}^{R}{\left\{\mathop{\sum }\limits_{i,j=1}^{M}{({l}_{i}{l}_{j}{p}_{ij}(r))}^{q}\right\}}^{K}.$$

The analytical derivation of the partition function allows us to calculate the mass exponent as $\tau (q)=\frac{{\rm{\log }}\,{Z}_{\epsilon }(q)}{{\rm{\log }}\,\epsilon }$, the generalized fractal dimension from $D(q)=\frac{\tau (q)}{1-q}$, the Lipschitz-Hölder exponent (also refer to coarse Hölder exponent or singularity index in prior work) $\alpha (q)=\frac{d\tau (q)}{dq}$ and the multifractal spectrum from *f*(*α*) = *α*(*q*)*q* − *τ*(*q*).

**Figure 2:**
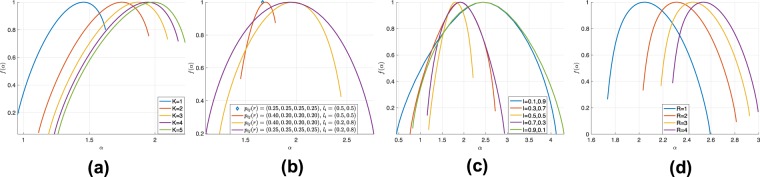
(**a**) Multifractal spectrums with *K* = 1…5, *M* = 2, *R* = 3, *p*_*ij*_(*r*) and *l*_*i*_ remain the same. (**b**) Multifractal spectrums with four different sets of initial unit square model parameters. *M* = 2, *R* = 3, *K* = 4. (**c**) Multifractal spectrums with $\left\{{l}_{i}\right\}=(0.1,0.9),(0.3,0.7),(0.5,0.5),(0.7,0.3),(0.9,0.1)$. *K* = 5, *M* = 2, *R* = 3, *p*_*ij*_(*r*) remains the same. (**d**) Multifractal spectrums with *R* = 1…4.

The networks generated by the proposed WMG model inherit a rich variety of multifractality compared with the unweighted MFNG model^32^. For example, when *R* = 1 (corresponding to MFNG model), the partition function in equation (3) shows that the variation in *K* does not influence significantly the mass exponent *τ*(*q*), the generalized multifractal dimension *D*(*q*), the Lipschitz-Hölder exponent *α*(*q*) and the multifractal spectrum *f*(*α*) because of the log operation. However, as shown in Fig. 2(a), for M = 2, R = 3 and same {*p*_*ij*_(*r*)} and {*l*_*i*_}, the variation in the model iteration step K causes a shift of multifractal spectrum towards a support on higher Lipschitz-Hölder exponents. One can observe that the shift in the support of the multifractal spectrum is smaller with increasing changes *ΔK* in the model iteration steps K.

The parabola-like curve shows the multifractality of the WMG model. Fig. 2(b) presents more results of multifractal spectrum curves with four different sets of initial unit square model parameters ${\left\{{p}_{ij}(r)\right\}}_{i,j=1:M,r=1:R}$ and ${\left\{{l}_{i}\right\}}_{i=1:M}$. Here, we consider *M* = 2, *R* = 3 and *K* = 4. The blue diamond is generated by equally assigning model parameters *p*_*ij*_(*r*) = (0.25, 0.25, 0.25, 0.25), *l*_*i*_ = (0.5, 0.5). The red curve corresponds to assigning *p*_*ij*_(*r*) = (0.40, 0.20, 0.20, 0.20), *l*_*i*_ = (0.5, 0.5). The yellow curve corresponds to *p*_*ij*_(*r*) = (0.40, 0.20, 0.20, 0.20), *l*_*i*_ = (0.2, 0.8). The purple curve is given by *p*_*ij*_(*r*) = (0.25, 0.25, 0.25, 0.25), *l*_*i*_ = (0.2, 0.8). Each set of parameters are the same for every weight layer *r* = 1…*R*.

Of note, for identical parameters (i.e. *p*_*ij*_(*r*) = (0.25, 0.25, 0.25, 0.25)), the WMG generates a monofractal network (represented in Fig. 2(b) by a blue diamond marker). More precisely, this implies that a more evenly assigned *p*_*ij*_(*r*) leads to more symmetric and narrower curve of the multifractal spectrum (which corresponds to a point for identical *p*_*ij*_(*r*)). A shift and reshape of the curve can also be caused by changing *l*_*i*_.

We observe that there is an interesting effect of the sub-interval length {*l*_*i*_} on the multi-fractal spectrum *f*(*α*). For example, as presented in Fig. 2(c), when node generating probabilities are identical ($\left\{{l}_{i}\right\}=(0.5,0.5)$ - yellow curve), the maximum value of multifractal spectrum *f*(*α*) places at leftmost compared with other curves given by asymmetric $\left\{{l}_{i}\right\}$. While interchanging the $\left\{{l}_{i}\right\}$ (i.e., $\left\{{l}_{i}\right\}=(0.3,0.7)$ - red vs. $\left\{{l}_{i}\right\}=(0.7,0.3)$ - magenta, or $\left\{{l}_{i}\right\}=(0.1,0.9)$ - blue vs. $\left\{{l}_{i}\right\}=(0.9,0.1)$ - green), the maximum value of the multifractal spectrum remains unchanged but the left and right hand side end points of the spectrum change. Similarly, the influence of {*p*_*ij*_(*r*)} can also be explored.

Fig. 2(d)  shows the influence of the weight resolution *R*. For different *R*, we assign the parameters ${p}_{ij}(r)=\frac{{c}_{ij}}{R}$, *r* = 1…*R*, where $${c}_{ij}=\left[\begin{array}{cc}0.25 & 0.29\\ 0.29 & 0.17\end{array}\right]$$. Therefore, the probability that an edge exists ${\sum }_{r=1}^{R}{p}_{ij}^{(K)}(r)$ remains the same among different *R*. It shows that increasing weight resolution *R* causes a right shift of multifractal spectrum.

### Statistical properties

To provide a first order characterization of the heterogeneity of complex weighted networks, we analyze the generalized degree ${\left\{{D}_{r}\right\}}_{r=1\ldots R}$ of a node, which quantifies the number of connections (edges) with an associated weight *w*_*r*_. The generalized degree distribution ${p}_{r}({k}_{r})=P\left\{{D}_{r}={k}_{r}\right\}$ retains more information of the network topology compared to the straightforward strength distribution, which helps us to better understand the network structure and its multifractality.

Via the extension of the multifractal network generator^32^, in the WMG model, the generalized degree distribution is expressed as

4 $${p}_{r}({k}_{r})=\mathop{\sum }\limits_{i=1}^{{M}^{K}}{p}_{i,r}({k}_{r}){l}_{i}^{(K)},$$

where  *p*_*i*,*r*_(*k*_*r*_) is the generalized degree distribution of nodes falling into interval *i*, ${p}_{i,r}({k}_{r})\approx poisson(N{\sum }_{j=1}^{{M}^{K}}{p}_{ij}^{(K)}(r){l}_{j}^{(K)})$. Detailed derivation of Eq. (4) is provided in Methods section ‘Generalized degree distributions’.

**Figure 3:**
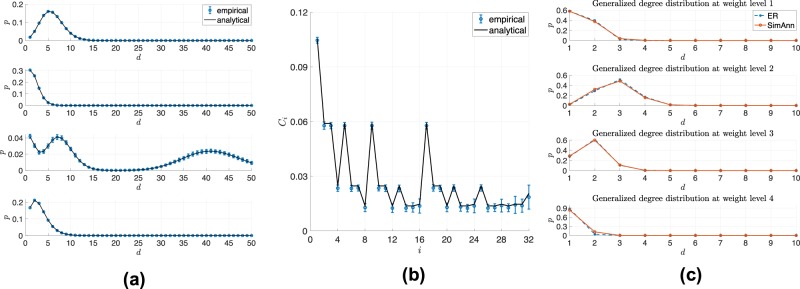
(**a**) Empirical and analytical generalized degree distribution. (**b**) Empirical and analytical results of clustering coefficients. This simulation is generated under parameters *M* = 2, *K* = 5, *R* = 4. The scale (number of nodes) of graph *N* = 5000. Graph generation is repeated 100 times. (**c**) The WMG model can represent networks generated by ER model.

 Fig. 3(a) illustrates the comparison between the analytical and empirical generalized degree distribution. The comparison analysis considers 100 runs, where networks of size N = 5000 nodes are generated from a set of random parameters {*p*_*ij*_(*r*)} and {*l*_*i*_}, with *M* = 2, *K* = 5, and *R* = 4. As one can notice, the WMG model can generate a wide variety of generalized degree distributions.

The clustering coefficients measure the local cliquishness and neighborhood connectivity^5^. Concerned with the density of connected triangles, it reflects the extent to which the neighbors of a certain node are also connected. In weighted graphs, the clustering coefficient of a node can be computed as the mean weights of triangles containing the node, divided by the mean weights of triplets (link pairs) originating from that node^42^. Following the derivation in the work of Palla *et al*.^32^, the average local clustering coefficient of a node falling into interval *i* can be calculated as

5 $${c}_{i}=\frac{{\sum }_{j,q=1}^{{M}^{K}}{\sum }_{{r}_{1},{r}_{2},{r}_{3}=1}^{R}\widehat{{w}_{1}}{l}_{j}^{(K)}{l}_{q}^{(K)}{p}_{ij}^{(K)}({r}_{1}){p}_{iq}^{(K)}({r}_{2}){p}_{jq}^{(K)}({r}_{3})}{{\sum }_{j,q=1}^{{M}^{K}}{\sum }_{{r}_{1},{r}_{2}=1}^{R}\widehat{{w}_{2}}{l}_{j}^{(K)}{l}_{q}^{(K)}{p}_{ij}^{(K)}({r}_{1}){p}_{iq}^{(K)}({r}_{2})},$$

where $\widehat{{w}_{1}}=\frac{w({r}_{1})+w({r}_{2})+w({r}_{3})}{3}$ and $\widehat{{w}_{2}}=\frac{w({r}_{1})+w({r}_{2})+w({r}_{3})}{3}$ are the arithmetic mean of triangles and triplets edge weights. Index *i* marks the interval which the node falls into when generating the graph. *i* = 1…*M*^*K*^. Comparison between empirical and analytical clustering coefficients is shown in Fig. 3(b). For most of the intervals, the empirical result fits well with the analytical one. However, smaller clustering coefficient corresponds with sparser local graph structure, thus standard deviation tends to be larger than the ones with comparative larger clustering coefficients.

The proposed *WMG* model can reproduce the statistical properties of classic theoretical models. We test our model on a weighted graph generated by the Erdős-Rényi model. The weighted version of Erdős-Rényi model is given by a set of linking probabilities ${\left\{p(r)\right\}}_{r=1:R}$, where *p*(*r*) is the corresponding linking probability with the discrete weight *w*(*r*). We apply the *WMG* model to a network with 500 nodes generated by a 4-layer Erdős-Rényi model. Fig. 3(c) illustrates the generalized degree distribution of the simulated network and the recovered WMG model. The blue asterisks denote the target distribution from the simulated ER graph, while the red circles represent the generalized degree distribution obtained from the identified WMG model. It shows that the proposed *WMG* model can capture the statistical properties of the classic random graph model.

### Multifractality in biological systems

**Figure 4:**
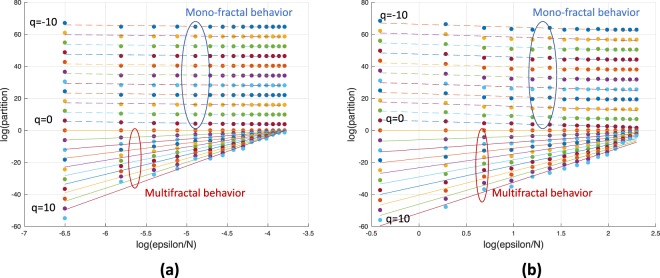
(**a**) Mono-fractal and multifractal scaling behavior in brain networks of cognitive normal person. (**b**) Mono-fractal and multifractal scaling behavior in chromosome contact maps of exponentially growing genome.

In two recent works^18,43^, the authors show that the interactions among different chromosomal regions and brain networks display a non-trivial multifractal property. We also applied the box-covering method proposed in^18^ to show the existence of multifractality in real networks. The box covering algorithm returns the mass of each boxes with radius *ϵ* to fully cover the network with *N* nodes. As illustrated in Fig. 4(a,b), the plots of partition functions and the scales reveal the multifractality behavior in the brain networks and the chromosome networks.

However, as discussed in section ‘Multifractal Analysis’, finding boxes to cover the graph is as hard (from a computational complexity perspective) as graph coloring problem and analytical expressions cannot be introduced. By mapping the real-world networks to multifractal generating measures that allow to construct weighted graph models, it allows us to characterize the complexity of real networks in function level and also makes it possible to better understand and control the topology structure. In this work, instead of counting boxes and calculating the multifractality in a given graph, we consider the multifractality in the recovered generating measures and its corresponding graph model.

Therefore, we exploit the proposed WMG model for investigating two biological systems, namely the chromosome interactions within the yeast genome and brain functionality networks during various stages of Alzheimer’s disease. Our goal is not only to characterize the generalized degree distributions in these real biological networks, but also to exemplify how a new network wide control strategy can be employed for ensuring specific mathematical network characteristics.

#### Yeast genome

Capturing the chromatin interactions via the Hi-C technique allows us to study the three-dimensional structure of the genome and understanding their observed behavior^44–46^. For instance, significant topological reorganization of yeast cell in the quiescence state has been observed^47^. Starting from these premises, we consider the yeast chromosome interaction data from Rutledge *et al*.^47^, where the Hi-C experiments are conducted on yeast cells in exponential growth on glucose-containing medium and on yeast cells in quiescence induced by glucose starvation. The data of Yeast genome are from the library of Juicebox software^44,45^. By interpreting the Hi-C matrix as an adjacency matrix corresponding to a complex weighted network, we estimate the WMG model from the yeast chromosome interaction data for both the exponential growth and quiescence states. We then regulate the multifractal spectrum of yeast cell in quiescence and transit it to the state of exponential growth using the proposed multifractality control strategy (see Methods section on ‘Multifractal control’).

**Figure 5:**
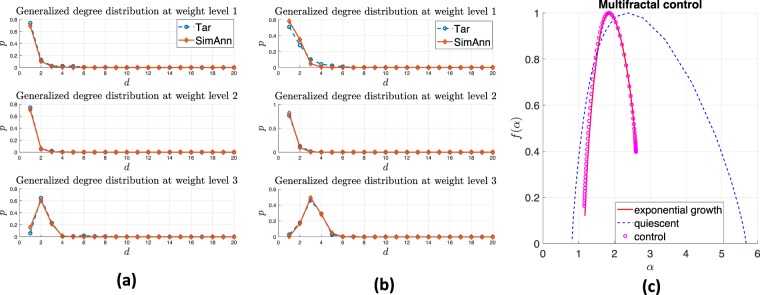
Case study: controlling yeast cell activity condition. (**a**) Generalized degree distributions of yeast cell Hi-C data in exponential growth. (**b**) Generalized degree distributions of yeast cell Hi-C data in quiescence. (**c**) Controlling multifractality of yeast cell in quiescent condition to exponentially growing condition.

 Fig. 5(a,b) illustrate the generalized degree distribution for the yeast cells in the exponential growth state and quiescence state, respectively. The blue asterisks denote the target distribution from chromosome interaction matrix, while the red circles represent the generalized degree distribution obtained from the identified WMG model. The simulated annealing algorithm can reproduce the model with similar statistical properties (in terms of shape and trend) to the real-world data (see Methods section on ‘Reconstructing weighted multifractal model’). Eq. (16) can also be redefined with any other desired network structure properties such as strength distribution and average nearest neighbour strength, but this is left for future work.

As discussed in Methods section ‘Multifractal control’, by solving Eq. (15), we modulate quiescent yeast cell to the desired state which yeast cell is growing exponentially. In Fig. 5(c), the red line represents the desired multifractal spectrum from actively growing cell. The curve with blue dots is the multifractal spectrum of quiescent yeast cell. The magenta asterisks are the optimal modulated solution given by simulated annealing algorithm. By controlling real-world network multifractality, we could enforce or track cell evolution procedure and therefore regulate bio-feature mechanisms yeast growth.

#### Alzheimer’s disease

Alzheimer’s disease (AD) is a neurodegenerative disease that leads to progressive cognitive decline. While it’s widely known that no cure exists for AD or terminating the neurodegeneration, early medical treatment might help to relieve the symptoms and slow the deterioration. To investigate and exemplify the benefit of proposed formalism within the context of this problem, we use data from the Alzheimer’s Disease Neuroimaging Initiative (ADNI) database (adni.loni.usc.edu). The rfMRI subject data are processed by BrainSuite fMRI Pipeline (BFP) and grayordinate representations are generated^48,49^.

The cerebral cortex is modeled as a surface mesh and globular subcortical nuclei are modeled as volume parcels^50^. The grayordinate data were downsampled to 445 nodes. The Pearson correlation matrix was computed using fMRI time-series^51^.

**Figure 6:**
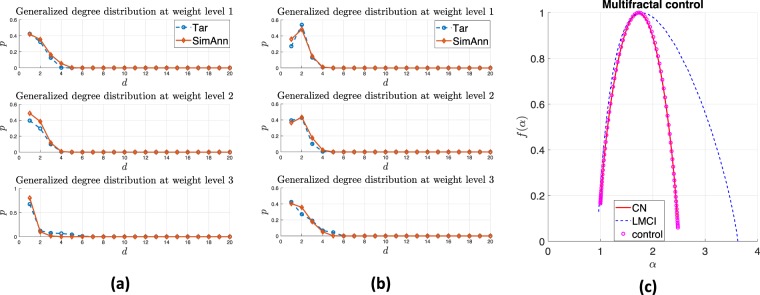
Case study: controlling brain structure of patients with Alzheimer’s disease. (**a**) Generalized degree distributions of cognitively normal (CN) cohorts brain interactions. (**b**) Generalized degree distributions of late mild cognitive impairment (LMCI) cohorts brain interactions. (**c**) Controlling multifractality of LMCI and modify it to CN.

To control the brain interactions matrix from late mild cognitive impairment (LMCI) patients and improve their brain structure, we apply our model and modulate it’s multifractality to fit the one from cognitively normal (CN) people. We follow the same procedure as discussed in Hi-C case study. Fig. 6(a,b) illustrate the generalized degree distributions of CN matrix and LMCI matrix, respectively. The blue asterisks represent the target distribution from CN/LMCI matrix and the red circles are the distribution of recovered model via the simulated annealing-based reconstruction algorithm (see section ‘Reconstructing weighted multifractal model’ in Methods). Simulated annealing (SA) algorithm provides a probabilistic approach for searching a large discrete space and finding approximate global optimum (e.g., parameters of a model) for a nonconvex optimization problem within a limited amount of time. Although it does not return the global optimum and it might not return identical results when running the SA algorithm multiple times, we find that one can distinguish between different networks from the retrieved WMG parameters. We individually run the simulated annealing algorithm on CN and LMCI matrix 200 times. We use 150 results of CN and 150 results of LMCI to train an SVM classifier with linear kernel, and using the remaining 50 of each class to test the dataset. The classification accuracy on the test dataset is 94%. It shows that the WMG model characterizes the network patterns and it could also discriminate between different traits of these complex networks.

Fig. 6(c)  shows the multifractal spectrum of LMCI matrix before and after the control. The red curve is the desired multifractal spectrum from CN data. The blue dots and magenta asterisks represent the multifractal spectrum curves of LMCI and optimal regulated solution obtained by adapting the LMCI network structure. The controlled multifractal spectrum also fits well with the target curve.

## Discussion

We propose the *weighted multifractal graph* (WMG) model to capture the nodes attributes and interactions in weighted complex networks. We show that it fits a wide variety of multifractal spectrum and statistical properties of real-world networks including generalized degree distribution, clustering coefficients and joint degree distribution. Beyond the self-similar constructing procedure of the recursive generating model, we present the multifractal analysis of the random graph model and show that the WMG model could generate a variety of multifractal spectrum curves with different shapes and varying degree of asymmetry. More importantly, the introduction of weights in the WMG model brings flexibility in multifractal spectrum. The proposed model has potential applications in many disciplines including biological systems, geoscience, financial markets and social networks. In order to ground this extended model into real-world applications, one needs to develop rigorous, efficient and accurate model identification techniques that need to take into account the relationship between the complexity of real networks and the generating measures, assess the impact of model parameters on network properties (e.g., information flow performance, robustness), all of which are not trivial mathematical tasks.

Apart from modelling real networks, an important implication of the WMG model is to minimally control the multifractality of complex networks. The experiments on real-world datasets of yeast genome and Alzheimer’s disease reveal that it is possible to regulate the multifractal spectrum of complex networks with minimal adjustments in the WMG model parameters. The proposed model is a step towards exploring the underlying growing mechanism of evolving networks such as brain structure of Alzheimer’s disease patients, or actively growing yeast cells. Moreover, the control of multifractality can provide a novel way to treat brain diseases, control growing states or recover from potential attacks in biological and social systems.

Future extensions of the WMG model can consider the generalization of *l*_*i*_. In the proposed WMG model, we consider that the values of the interval length *l*_*i*_ are identical for all *R* layers. However, one can consider that *l*_*i*_ depends on *r*, but this will introduce more complexity and model identification strategies. Moreover, each node can have different category *i*_*r*_ in different weight layer *r* and the linking probability would become ${p}_{{i}_{r}{j}_{r}}^{(K)}(r)$. Another important addition would be to have temporal dimension into the proposed model. This corresponds to modelling and controlling the time varying complex networks by regulating the multifractality of graphs. It will help to answer research questions such as how does network structure evolve over time from one state to another. For example, in decoding human behavior, what are the causes of changing genome or brain structure and what does it lead to. It is also crucial to understand the physical meaning of network multifractality and deciphering the hidden information of real-world complex networks with self-similar patterns.

## Methods

### Multifractal analysis

We study the multifractal analysis of the WMG model based on the partition function. Let ${\left\{{p}_{ij}(r)\right\}}_{i,j=1:M}$ denote links generating probabilities in the initial unit square model. For simplicity, we reshape it as ${\left\{{p}_{i}(r)\right\}}_{i=1:{M}^{2}}$. ${\left\{{l}_{i}{l}_{j}\right\}}_{i,j=1:M}$ is the area of each sub-rectangles in the unit square. Similarly, we reshape it as ${\left\{{a}_{i}\right\}}_{i=1:{M}^{2}}$. The partition function at an average sub-block size $\epsilon =\frac{1}{R}{\left(\frac{1}{M}\right)}^{2K}$ can be defined as

6 $${Z}_{\epsilon }(q)=\sum _{r,{\left\{{k}_{j}\right\}}_{j=1:{M}^{2}}}\left(\begin{array}{c}K\\ {k}_{1}\ldots {k}_{j}\ldots {k}_{{M}^{2}}\end{array}\right){\left\{\mathop{\prod }\limits_{i=1}^{{M}^{2}}{[{a}_{i}{p}_{i}(r)]}^{{k}_{i}}\right\}}^{q}.$$

Here the first term $\left(\genfrac{}{}{0.0pt}{}{K}{{k}_{1}\ldots {k}_{j}\ldots {k}_{{M}^{2}}}\right)$ is number of sub-blocks which have the same area proportion ${\prod }_{i=1}^{{M}^{2}}{{a}_{i}}^{{k}_{i}}$ and link generating probability ${\prod }_{i=1}^{{M}^{2}}{{p}_{i}(r)}^{{k}_{i}}$. The second term ${\prod }_{i=1}^{{M}^{2}}{[{a}_{i}{p}_{i}(r)]}^{{k}_{i}}$ is the proportion of edges with weight *w*_*r*_ which are generated by those sub-blocks. ${\left\{{k}_{j}\right\}}_{j=1:{M}^{2}}$ is subjected to ${\sum }_{j=1}^{{M}^{2}}{k}_{j}=K$. We continue to simplify the summation and multinomial coefficients.

7 $$\begin{array}{ll}{Z}_{\epsilon }(q) & \mathop{=}\limits^{A}\sum _{r,{\{{k}_{j}\}}_{j=3:{M}^{2}}}\mathop{\prod }\limits_{i=3}^{{M}^{2}}{[{a}_{i}{p}_{i}(r)]}^{q{k}_{i}}\left(\genfrac{}{}{0.0pt}{}{K}{{k}_{3}\ldots {k}_{{M}^{2}}}\right)\sum _{{k}_{2}}\left(\genfrac{}{}{0.0pt}{}{K-{k}_{3}\cdots -{k}_{{M}^{2}}}{{k}_{2}}\right){[{a}_{1}{p}_{1}(r)]}^{q{k}_{1}}\cdot {[{a}_{2}{p}_{2}(r)]}^{q{k}_{2}}\\  & \mathop{=}\limits^{B}\sum _{r,{\{{k}_{j}\}}_{j=3:{M}^{2}}}\mathop{\prod }\limits_{i=3}^{{M}^{2}}{[{a}_{i}{p}_{i}(r)]}^{q{k}_{i}}\left(\genfrac{}{}{0.0pt}{}{K}{{k}_{3}\ldots {k}_{{M}^{2}}}\right){\{{[{a}_{1}{p}_{1}(r)]}^{q}+{[{a}_{2}{p}_{2}(r)]}^{q}\}}^{K-{k}_{3}\cdots -{k}_{{M}^{2}}}\\  & \mathop{=}\limits^{C}\mathop{\sum }\limits_{r=1}^{R}{\left\{\mathop{\sum }\limits_{i=1}^{{M}^{2}}{{a}_{i}{p}_{i}(r)}^{q}\right\}}^{K}=\mathop{\sum }\limits_{r=1}^{R}{\left\{\mathop{\sum }\limits_{i,j=1}^{M}{({l}_{i}{l}_{j}{p}_{ij}(r))}^{q}\right\}}^{K}.\end{array}$$

In equation (7), the partition function is further simplified with binomial theorem and multinomial coefficients properties. Continuing with Eq. (6), the first equal label $\mathop{=}\limits^{A}$ is given by expanding multinomial coefficient $\left(\genfrac{}{}{0.0pt}{}{K}{{k}_{1}\ldots {k}_{j}\ldots {k}_{{M}^{2}}}\right)$. *B* uses the constraint of ${\sum }_{i=1}^{{M}^{2}}{k}_{j}=K$. Further simplifications are continued on ${k}_{3},{k}_{4}\ldots {k}_{{M}^{2}}$ with mathematical induction (*C*).

Other functions and measures including the mass exponent $\tau (q)=\frac{{\rm{\log }}\,{Z}_{\epsilon }(q)}{{\rm{\log }}\,\epsilon }$, the generalized fractal dimension $D(q)=\frac{\tau (q)}{1-q}$, the Lipschitz-Hölder exponent (also refer to coarse Hölder exponent or singularity index in some materials) $\alpha (q)=\frac{d\tau (q)}{dq}$ and the multifractal spectrum *f*(*α*) = *α*(*q*)*q* − *τ*(*q*) can be derived from the partition function in equation (7). Here we give the expression of the Lipschitz-Hölder exponent.

8 $$\alpha (q)=\frac{1}{{Z}_{\epsilon }(q){\rm{\log }}\,\epsilon }\mathop{\sum }\limits_{r=1}^{R}\left\{K{\{\mathop{\sum }\limits_{i,j=1}^{M}\left.{[{l}_{i}{l}_{j}{p}_{ij}(r)]}^{q}\right)\}}^{K-1}\mathop{\sum }\limits_{i,j=1}^{M}{[{l}_{i}{l}_{j}{p}_{ij}(r)]}^{q}{\rm{ln}}\,[{l}_{i}{l}_{j}{p}_{ij}(r)]\right\}.$$

### Generalized degree distribution

We introduce the generalized degree distribution to specify the network structure with more detailed information compared to the strength distribution. Generalized degree *D*_*r*_ is defined as the number of edges or connections a node has with weight *w*_*r*_. When generating the nodes, we classify each node into *M*^*K*^ categories with probability ${l}_{i}^{(K)}$, *i* = 1…*M*^*K*^. Given by the recursive model, the link generating probability of a node in category *i* and a node in category *j* is ${p}_{ij}^{(K)}(r)$ and the edges are generated independently. Thus, the number of edges connecting a node in category *i* and all nodes in category *j* follows binomial distribution. As discussed in the publication of Palla *et al*.^32^, when the network scale *N* is large, it can be further approximated by Poisson distribution with rate ${\lambda }_{i,j}(r)={p}_{ij}^{(K)}(r)N{l}_{j}^{(K)}$. Followed by summation property of Poisson distributed independent random variables, the generalized degree distribution of a node falling into interval *i* is given by

9 $${p}_{i,r}({k}_{r})\approx Poisson(\mathop{\sum }\limits_{j=1}^{{M}^{K}}{\lambda }_{i,j(r)},{k}_{r})=Poisson(N\mathop{\sum }\limits_{j=1}^{{M}^{K}}{p}_{ij}^{(K)}(r){l}_{j}^{(K)},{k}_{r}).$$

Therefore, the average generalized degree distribution is expressed as

10 $${p}_{r}({k}_{r})=\mathop{\sum }\limits_{i=1}^{{M}^{K}}{p}_{i,r}({k}_{r}){l}_{i}^{(K)}.$$

As shown in the main text, the analytic expression in (10) coordinates well with empirical simulation results when *N* ≫ *M*^*K*^.

### Clustering coefficients

Clustering coefficient is a measure of graph cliquishness and neighborhood connectivity^5^. Following the definition of clustering coefficients in weighted graphs^42^ and the multifractal network generator^32^, the clustering coefficient of a node in category *i* is written as

11 $${c}_{i}=\frac{{\sum }_{j,q=1}^{{M}^{K}}{\sum }_{{r}_{1},{r}_{2},{r}_{3}=1}^{R}\widehat{{w}_{1}}{l}_{j}^{(K)}{l}_{q}^{(K)}{p}_{ij}^{(K)}({r}_{1}){p}_{iq}^{(K)}({r}_{2}){p}_{jq}^{(K)}({r}_{3})}{{\sum }_{j,q=1}^{{M}^{K}}{\sum }_{{r}_{1},{r}_{2}=1}^{R}\widehat{{w}_{2}}{l}_{j}^{(K)}{l}_{q}^{(K)}{p}_{ij}^{(K)}({r}_{1}){p}_{iq}^{(K)}({r}_{2})},$$

where $\widehat{{w}_{1}}=\frac{w({r}_{1})+w({r}_{2})+w({r}_{3})}{3}$ and $\widehat{{w}_{2}}=\frac{w({r}_{1})+w({r}_{2})+w({r}_{3})}{3}$ are the arithmetic mean of triangles and triplets edge weights. The numerator is the mean weights of triangles containing a node falling into sub-interval *i*, *i* = 1…*M*^*K*^, the denominator is the mean weights of triplets (link pairs) originating from that node.

### Joint degree distribution

The joint degree distribution of graphs with discrete weight set *w*(*r*) is defined as $p({d}_{1},{d}_{2},r)=\frac{m({d}_{1},{d}_{2},r)}{M(r)}$, where *M*(*r*) is the total number of edges with weight *w*(*r*), *m*(*d*_1_, *d*_2_, *r*) is the number of edges connecting nodes with the generalized degree *d*_1_ and *d*_2_, and the weight is *w*(*r*). The number of nodes which have *d* edges with weight *w*(*r*) is:

12 $${N}_{i}(d,r)=N{l}_{i}\times Poisson(\mathop{\sum }\limits_{j=1}^{{M}^{K}}{p}_{ij}^{(K)}(r)N{l}_{j}^{(K)},d).$$

Therefore, we could obtain the expression of the joint degree distribution as

13 $$\begin{array}{lll}p({d}_{1},{d}_{2},r) & = & \frac{{\sum }_{i,j=1}^{{M}^{K}}{N}_{i}({d}_{1},r){N}_{j}({d}_{2},r){p}_{ij}^{(K)}(r)}{{\sum }_{i,j=1}^{{M}^{K}}N{l}_{i}^{(K)}N{l}_{j}^{(K)}{p}_{ij}^{(K)}(r)}.\end{array}$$

**Figure 7:**
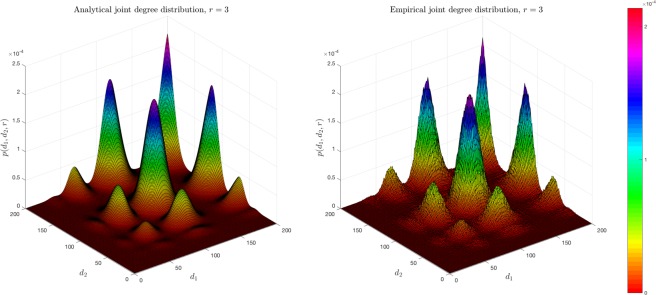
Analytical and empirical joint degree distribution. This simulation is generated under parameters *M* = 2, *K* = 3, *R* = 4, *N* = 5000. Graph generation is repeated 10 times.

The comparison between the analytical and empirical joint degree distribution is shown in Fig. 7.

### Edge distribution

In the WMG model, we focus on weighted complex networks. Therefore, we also consider the edge distribution in the proposed model. Let *X*_*ij*_(*r*) denote the number of edges connecting nodes in category *i* and nodes in category *j* with weight *w*(*r*). The vector of edge numbers **X**_**ij**_ follows the multinomial distribution $Multi({p}_{ij}^{(K)}(0),{p}_{ij}^{(K)}(1),\ldots ,{p}_{ij}^{(K)}(R))$. The total number of edges with weight *w*(*r*) is the sum of the independent random variables $X(r)={\sum }_{ij}^{{M}^{K}}{X}_{ij}(r)$. Its moment generating function can be written as

14 $$MG{F}_{{\bf{X}}}({\bf{t}})=\mathop{\prod }\limits_{i,j=1}^{{M}^{K}}MG{F}_{{{\bf{X}}}_{{\rm{ij}}}}({\bf{t}})=\mathop{\prod }\limits_{i,j=1}^{{M}^{K}}{\left[\mathop{\sum }\limits_{r=0}^{R}{p}_{ij}^{(K)}(r){e}^{{t}_{r}}\right]}^{{N}^{2}{l}_{i}^{(K)}{l}_{j}^{(K)}}.$$

### Controlling multifractality

The proposed *WMG* model can quantify the emergence and self-organization^52^ and complexity of network in terms of number of rules that are at play when growing a network. Introducing the WMG model makes it possible to minimally control complex network. As discussed in previous section, by computing its partition function, multifractal spectrum and Lipschitz-Hölder exponent, we could analytically obtain network multifractalities. While aiming to transit or change a multifractal network to some certain networks with desired multifractality, we also expect minimal adjustments comparing with original network.

This control problem can be written as the following optimization

15 $$\begin{array}{lll}{\{{p}_{i,j}(r)\}}_{i,j,r},{\{{l}_{i}\}}_{i} & = & \,{\rm{\arg }}{{\rm{\min }}}_{p,l}\parallel f(p,l)-{f}_{Tar}{\parallel }_{2}+\parallel \alpha (p,l)-{\alpha }_{Tar}{\parallel }_{2}\\  &  & +\lambda (\parallel p-{p}_{0}{\parallel }_{2}+\parallel l-{l}_{0}{\parallel }_{2}),\end{array}$$

where *f*_*Tar*_, *α*_*Tar*_ are the target multifractal spectrum and the Lipschitz-Hölder exponent, *f*(*p*, *l*) and *α*(*p*, *l*) are given by proposed multifractal network model, ${\left\{{p}_{i,j}(r)\right\}}_{i,j,r},{\left\{{l}_{i}\right\}}_{i}$ are the optimal parameters solutions, and *p*_0_, *l*_0_ are the starting point of model parameters. To focus on the influence of minimal changes in model parameters, here we fix *M*, *R* and *K* to some certain integers. To minimize the cost function defined in equation (15), here we use simulated annealing algorithm^53^ to recover optimal model parameters ${\left\{{p}_{i,j}(r)\right\}}_{i,j,r}$ and ${\left\{{l}_{i}\right\}}_{i}$.

**Figure 8:**
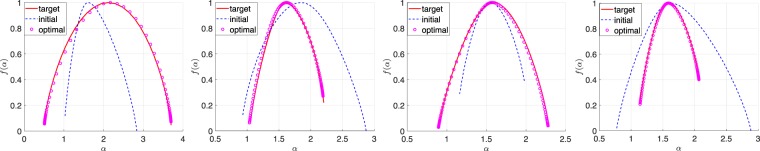
Multifractal control simulations with four different perturbations of target multifractal spectrum.

Four multifractal control problem simulations are shown in Fig. 8. The red lines are the desired multifractal spectrums, blue dot lines are the starting multifractal spectrums given by random parameters. Magenta asterisks are the optimal solutions given by simulated annealing algorithm. Different deviations of shifting and scaling are shown here. In each case, the multifractal spectrums are almost identical to the desired ones. It shows that by minimizing the cost function in equation (15), we could control network multifractality with minimal changes.

### Reconstructing weighted multifractal model

We use simulated annealing algorithm^53^ to retrieve the optimal parameters $\left\{{p}_{ij}(r)\right\}$ and $\left\{{l}_{i}\right\}$ in the real networks. We define the distance of statistical measures in the WMG model and the real networks as energy function,

16 $$E=\mathop{\sum }\limits_{r=1}^{R}\mathop{\sum }\limits_{d=1}^{N}\parallel p(d,r)-{p}^{* }(d,r){\parallel }_{2}+\lambda \parallel \langle {c}_{i}\rangle -{c}^{* }{\parallel }_{2},$$

where p^*^(*d*, *r*) and *p*(*d*, *r*) are the generalized degree distributions generated from the real-world network and the proposed WMG model by equation (4), $\left\langle {c}_{i}\right\rangle$ is the average clustering coefficient in equation (5), c^*^ is the empirical clustering coefficient. Equation (16) could also be redefined by other desired statistical properties of the networks. Minimizing Eq. (16) is a non-convex problem. And as the weight resolution in the WMG model increases, more parameters need to be estimated. Other optimization methods may also be applied.
